# Nutritional Characteristics of Four Underutilized Edible Wild Fruits of Dietary Interest in Ghana

**DOI:** 10.3390/foods8030104

**Published:** 2019-03-20

**Authors:** Matthew Atongbiik Achaglinkame, Ruth Olaide Aderibigbe, Oliver Hensel, Barbara Sturm, Joseph Kudadam Korese

**Affiliations:** 1 Faculty of Agriculture, Department of Agricultural Mechanization and Irrigation Technology, University for Development Studies, P.O. Box TL 1882, Nyankpala Campus, Tamale, Ghana; maachaglinkame@gmail.com; 2 Product Development Programme, National Horticultural Research Institute, Jericho G.R.A., Ibadan 200272, Oyo State, Nigeria; connecttolaide@yahoo.com; 3 Faculty of Organic Agricultural Sciences, Section of Agricultural and Biosystems Engineering, University of Kassel, Nordbahnhofstraße 1a., 37213 Witzenhausen, Germany; agrartechnik@uni-kassel.de (O.H.); barbara.sturm@uni-kassel.de (B.S.)

**Keywords:** malnutrition, underutilized, wild forest fruits, nutritional value

## Abstract

Malnutrition has been a serious issue in Ghana and Africa as a whole. However, the potential of many indigenous fruits to combat it has not yet been tested. Therefore, this study aimed to determine the nutritional characteristics of four underutilized wild fruits (*Gardenia* *erubescens*, *Sclerocarya birrea, Diospyros mespiliformis*, and *Balanites aegyptiaca*) of dietary interest in Ghana. The nutritional and antinutritional characteristics of the fruits were analyzed according to standard methods (laid down by the Association of Official Analytical Chemists and other well-known researchers) on a dry weight (dw) basis. The nutritional value of the fruits was high enough to contribute to the nutrient requirements of humans, with their iron (0.34–1.46 mg/100 g), zinc (0.81–2.97 mg/100 g), vitamin A (0.84–2.03 mg/100 g), and β-carotene (64.84–176.89 mg/100 g) contents worth special mention. The antinutrient content also ranged between 0.06–1.82 mg/g. Therefore, it is evident from the study that the fruits, although containing some levels of antinutrients, are nutrient-dense, suggesting their potency in fighting malnutrition in humans.

## 1. Introduction

Underutilized edible wild fruits have become a very important part of human nutrition and cannot be overlooked as far as food security, good health, and income generation are concerned [1,2]. These wild fruits provide humans with very essential nutrients such as dietary fiber, protein, and sugars, as well as health-promoting phytochemicals and minerals [3]. Recent research has suggested that wild fruits have a curing ability for multiple disorders such as diabetes, cardiovascular problems, inflammations, and digestive and urinary tract disorders, among others, due to their rich fiber and antioxidant components [4,5,6]. Thus, food insecurity in developing countries can be reduced by motivating rural poor communities to increase their consumption of indigenous fruits as well as fruit-based food supplements. Accordingly, the Food and Agricultural Organization of the United Nations (FAO) is currently promoting the conservation and sustainable use of biodiversity for nutrition and agriculture as a means to increase dietary diversity [7,8,9]. However, the main problem relating to the full exploitation of some of these wild fruits is the presence of some antinutrients, which have the potential to reduce especially protein and starch digestibility and mineral bioavailability [10,11,12].

Many African countries including Ghana are greatly blessed with lots of fruit-bearing species, which yield both domesticated and wild fruits [2,13,14] at one point or another throughout the year. However, it is disappointing that nutrient deficiencies are still very common and alarming especially among children and pregnant women in the country. Statistics shows that about 66% of children aged 6–59 months and 42% women of reproductive age (15–49 years) in Ghana are anemic [15,16]. Furthermore, about 75% of young children and 20% of pregnant women in Ghana are vitamin A-deficient [17]. 

Research by Bvenura and Sivakumar [2] showed that many people in sub-Saharan Africa do not eat enough fruits, and this compromises the delivery of a balanced and healthy diet. In Ghana for example, about 56% of adolescents rarely (≤3 days per week) consumed fruits [18]. However, the World Health Organization (WHO) recommends a daily intake of more than 400 g of fruit per person to protect against diet-related non-communicable diseases [19]. Several factors have been opined to be the root causes of the low consumption, some of which include unavailability, unaffordability, ignorance, and neglect [20,21,22]. Ignorance and neglect may paramountly account for the low consumption of fruits in areas such as Ghana where nature generously supplies varieties of fruits all year round. Some foods are sometimes tagged as poor people’s foods [23], and thus are deliberately ignored. Lack of knowledge of the nutritional and health benefits of certain foods makes some people, the educated and uneducated alike, disregard these foods [24,25]. This may not be different in the case of Ghana following statistical evidence of nutrient deficiencies, despite the availability of both domesticated and wild fruits in the various parts of the country. The consumption of predominantly traditional starchy crops (cereals and root crops), especially by the rural poor, is also reported to impose both micronutrient and protein malnutrition [26].

To recommend wild fruits as a contribution to an improved diet, knowledge about the nutritional value is required. A food composition database is of great importance in addressing health and nutrition issues. According to Stadlmayr et al. [27], such a database is important for planning food, nutrition, and health-related policy tools. As a result, there is a worldwide call to develop a National Food Composition Database. Moreso, nutritional information is required in order to develop processing guidelines for the production of target, processed products that are suitable for low-income populations. Unfortunately, Ghana does not have a national food database of its own. 

Therefore, the current study was conducted to evaluate the nutritional characteristics of four (4) wild fruits, namely *Gardenia erubescens*, *Sclerocarya birrea, Diospyros mespiliformis,* and *Balanites aegyptiaca* from Ghana. Literature search has shown that the fruits of *G. erubescens*, *S. birrea, D. mespiliformis,* and *B. aegyptiaca* have been characterized through the fruit or pulp [28,29,30] and seeds [31,32] in some parts of the world. Studies have provided some promising nutritional information especially on the proximate, mineral compositions [28,29,30] and vitamin C content [28,29] of one or more of these fruits. Bello et al. [29] have also reported the presence of some antinutrients such as tannin, phytate, and oxalate in *G. erubescens* and other fruits in their study. While these data are relevant, information on these Ghanaian wild fruits is lacking. Furthermore, the soil and climatic conditions of different regions result in a significant difference in the food composition of the food that is produced, and therefore data cannot simply be borrowed between countries. Besides, information on other vital nutrients such as vitamin A, β-carotene, and other phytochemicals such as polyphenols and flavonoids is limited if not lacking, hence this study. The data in this study may be incorporated into food nutrient databases such as the FAO/INFOODS (International Network of Food Data Systems) databases, and also foster renewed interest in their use in contemporary diets, especially in Ghana. 

## 2. Materials and Methods 

### 2.1. Source of Raw Materials

Fruit samples (Table 1) were obtained from the wild in Wiaga (10°39′0″ N, 1°16′0″ W), in the Builsa North District of the Upper East region of Ghana. The fruits were taken from as many different plants of each type as could be accessed in the fresh state, except for *Diospyros mespiliformis*, which were in the dried state at the time of collection, because the fresh ones were out of season. The fruits were transported to the laboratory and stored at 4 °C prior to processing.

### 2.2. Sample Preparation and Processing

The fruits obtained were sorted and washed where applicable. Then, seeds and unwanted parts were removed manually with the aid of stainless steel knives, while only the edible portions of the fruit were used for the study. The pulp and peel of *G. erubescens*, *S. birrea,* and *D. mespiliformis* and pulp of *B. aegyptiaca* were the parts of the fruits that were used for the study. Then, the fruit samples were dried at 60 °C for 12 h in a hot-air oven, according to Afolabi [33]. The dried samples were milled using a Moulinex miller (MC300, Moulinex, Hong Kong, China) and stored in airtight Ziploc bags at 4 °C until analysis.

### 2.3. Chemical Analysis

#### 2.3.1. Proximate

The moisture, ash, protein, fiber, and fat contents of the samples were analyzed according to Association of Official Analytical Chemists (AOAC) procedures [34]. Carbohydrate content was calculated as the difference: 100 – (moisture (g) + protein (g) + fiber (g) + fat (g) + ash (g)) [34]. All analyses were performed in triplicate. The results were expressed in g per 100 g of dry weight (g/100 g dw). The energy content of each fruit was calculated as follows: Energy (kcal/ 100 g) = 4 × protein (g) + 4 × carbohydrates (g) + 9 × fat (g). 

#### 2.3.2. Determination of pH, Total Soluble Solids, and Titratable Acidity

Ten grams of each milled sample were dissolved in 100 mL of distilled water. Then, the pH was determined using a pH meter BASIC 20 manufactured by Crison Instruments (Alella, Spain) after the solution was stirred for 30 min and left to rest for 10 min. Total soluble solids (TSS) were determined using an Atago DR-A1digital refractometer (Atago Co. Ld., Tokyo, Japan) at 25 °C. Titratable acidity was determined on 10% slurry of each sample. The slurry was filtered, and 50 mL of the filtrate was transferred into a clean 250-mL beaker. Three drops of phenolphthalein indicator were added to the solution, and 0.1 M of NaOH was slowly titrated to an endpoint. Then, titratable acidity was calculated as percent citric acid as follows: Acidity (%) = (titre value (mL NaOH) × acid value (0.0064) × 100 mL)/(Sample Weight) × 50 mL.

#### 2.3.3. Mineral Determination

The digestion of the samples was done by wet ashing, and the calcium (Ca), potassium (K), magnesium (Mg), phosphorus (P), sodium (Na), iron (Fe), and zinc (Zn) contents of the fruits were quantified with a Flame Atomic Absorption Spectrophotometer (AAS Model Nov AA 400p) according to [34]. 

### 2.4. Vitamins (A, B3, C), β-Carotene, Total Polyphenols, and Flavonoids 

The vitamin A, B3, and C contents and the β-carotene content of the fruits were also determined following the procedures of [34]. The polyphenol content was determined according to the method described by Singleton et al. [35]. One gram of dried powdered sample of each of the fruits was soaked in 10 mL of methanol 70% (*w/v*) and centrifuged at 1000 rpm for 10 min. Then, one milliliter of the supernatant was oxidized with 1 mL of Folin-Ciocalteu’s reagent and neutralized by 1 mL of 20% (*w/v*) sodium carbonate afterwards. After incubating the reaction mixture for 30 min at ambient temperature, absorbance was measured at 745 nm using a spectrophotometer (PG Instruments, England). A calibration curve of gallic acid (1 mg/mL) as standard was used to obtain the polyphenol content. Each analysis was repeated thrice, and the average value was obtained. The same procedure was followed for the preparation of the standard solution of gallic acid. The total flavonoid content was determined according the method ascribed by Meda et al. [36]. Briefly, 0.5 mL of the methanolic extract was mixed with 0.5 mL of methanol, 0.5 mL of AlCl3 (10%, *w/v*), 0.5 mL of potassium acetate (1 M), and 2 mL of distilled water. The mixture was allowed to incubate at ambient temperature for 30 min. Thereafter, the absorbance was measured at 415 nm by using a spectrophotometer (PG Instruments, Woodway lane, UK). The total flavonoids were determined using a calibration curve of quercetin (0.1 mg/mL) as standard. This procedure was repeated three times for each sample, and their mean values were computed. The same procedure was followed for the preparation of the standard solution of quercetin. 

### 2.5. Antinutrient Determinations

Phytate content was determined using the Wade’s reagent colorimetric method [37]. One gram of the dried powdered sample was mixed with 20 mL of hydrochloric acid (0.65 N) and stirred for 12 h with a magnetic stirrer. The mixture was centrifuged at 12,000 rpm for 40 min. About 0.5 mL of supernatant was added to 3 mL of Wade’s reagent. After the reaction mixture was incubated for 15 min, absorbance was then taken at 490 nm with a spectrophotometer (PG Instruments). Phytate content was obtained using a calibration curve of sodium phytate (10 mg/mL) as standard. Oxalate content was determined using the titration method as outlined by Day and Underwood [38]. Tannin content was analyzed according to procedures established by Price et al. [39], while saponin content was determined according to Obadoni and Ochuko [40].

### 2.6. Statistical Analysis 

The data obtained from the study were analyzed by one-way analysis of variance (ANOVA) followed by Tukey’s studentized range test with significance set at *p* ≤ 0.05 in the Minitab^®^ 16.2.2 (Minitab Inc., State College, PA, USA) statistical tool, and the results are presented in scientific tables.

## 3. Results and Discussion

### 3.1. Proximate and Physicochemical Properties

The proximate and physicochemical properties of the four fruits are shown in Table 2. The highest moisture content was recorded in *S. birrea* (85.84%) while the least was recorded in *D. mespiliformis* (6%). The reverse was the case for dry matter; *D. mespiliformis* had the highest (93.99%), while *S. birrea* had the lowest (14.16%). These observations are comparable to those in the literature [29,41,42]. The relatively higher moisture contents in *S. birrea* and *G. erubescens* suggest shorter storability if left unprocessed within a reasonable period of time, hence a need for processing into more stable products. Considering the relatively high dry matter contents of the fruits, it may be economically feasible for food product development, since too much raw material may not be needed to give a quantifiable amount of total solids. 

The carbohydrate content averagely varied from 61.69% in *S. birrea* to 76.44% in *G. erubescens*, representing about 27–59% of the recommended dietary allowance (RDA) of carbohydrate (130–210 g/day) across all age groups [43]. This means that the fruits have a great potential to supply the human body with good amounts of its primary source of energy. Generally, high protein values were recorded among the fruits, with *S. birrea* having the highest values (12.48%), while *G. erubescens* had the lowest (8.83%). The protein value of *S. birrea* in this study is quite higher than that (8%) cited by Mateke [44]; likewise, that of *B. aegyptiaca* (9.19%) was also higher than that (6.39%) cited by Mateke, as reported by Admassu, Bekele, and Kim [28]. With these inherent protein values, these fruits may be able to contribute about 16–123% to the daily protein requirement, depending on the age group. 

The ash content of the fruits, which ranged from 3.00% in *D. mespiliformis* to 6.37% in *S. birrea*, is suggestive of their mineral power, and thus when consumed could play a significant role in curbing some micronutrient deficiencies troubling human lives. The ash value obtained for *S. birrea* was greater than the 4.9% reported by Amarteifio and Mosase [41], but similar to the 6.8% reported by Murray et al. [42]. However, the 4.4% recorded in this study for *B. aegyptiaca* is lower than the 5.66% reported by Admassu et al. [28]. 

The fat contents were averagely low except for *S. birrea*, whose value was relatively higher (9.68%). Low fat values could mean that the fruits may play an important role in curtailing cardiovascular problems. Nevertheless, the relatively high fat content of *S. birrea* may provide a feeling of satiety, and thus could help reduce hunger. A range of 2.00% in *D. mespiliformis* to 4.25% in *S. birrea* was recorded as crude fiber for the fruits, which represents about 5–22% of the RDA of fiber (19–38 g/day) for humans. This amount of fiber could be of immense importance in the digestive process of humans in controlling constipation. The energy content (kcal) of the fruits was observed to increase with their fat contents, ranging from 354.48 in *B. aegyptiaca* to 383.81 in *S. birrea*. This suggests the fruits could supply approximately 11–19% of the energy (2071–3152 kcal/day) requirement of the body when consumed. 

The pH values ranged from 4.20 in *S. birrea* to 5.44 in *D. mespiliformis*, indicating that the fruits are slightly acidic. However, the total soluble solid content of the fruits was low following a range of 1.01% in *G. erubescens* to 1.56% in *D. mespiliformis*. The titratable portions of the acid content of the fruits also varied from 1.04% in *G. erubescens* to 2.35% in *B. aegyptiaca*. The range for the titratable acidity in this study was similar to the 0.6–1.7% reported in *S. birrea* and other fruits elsewhere [31].

### 3.2. Mineral Composition 

Generally, it could be observed that the fruits have relatively high concentrations of the minerals analyzed (Table 3). The concentration of calcium ranged from 6.48 mg/100 g in *G. erubescens* to 14.95 mg/100 g in *S. birrea*. Calcium plays an important role in strong bone and teeth formation, the regulation of muscle contractions, and the transmission of nerve impulses in the body; thus, its presence in human diets is a necessity [45].

Potassium concentration averagely ranged from 129.4 mg/100 g in *D. mespiliformis* to 133.41 mg/100 g in *G. erubescens*. However, there was no statistically noticeable variation in potassium concentration among the fruits (*p >* 0.05). Potassium plays a very crucial role in the body by helping maintain body fluid and osmotic balance, as well as aiding in the regulation of nerve signals and muscle contractions [46,47]. Phosphorus values, on the other hand, ranged significantly (*p <* 0.05) from 46.37 mg/100 g in *G. erubescens* to 103.87 mg/100 g in *S. birrea*. The role of phosphorus in the formation of strong bones and teeth, the maintenance of a regular heartbeat, muscle contraction, regulation of the storage and use of body energy, among other roles, cannot be undervalued. 

A range of 162.98 mg/100 g in *D. mespiliformis* to 177.69 mg/100 g in *S. birrea* of magnesium was observed among the fruits, with each varying significantly (*p <* 0.05) from the other. It has been established that magnesium helps relax muscles along the respiratory pathway, enabling asthmatics to breathe with ease [29]. Its role in most phosphate transfer reactions, the structural stability of nucleic acid, and the intestinal absorption of nutrients also cannot be underestimated. Its deficiency has been linked with problems such as hypertension, stroke, severe diarrhea, and migraines, to mention a few [48]; however, the consumption of these fruits may help prevent these health issues, following their high values. 

For sodium content, *G. erubescens* recorded the highest value (2.64 mg/100 g), while *D. mespiliformis* recorded the lowest (1.25 mg/100 g). Sodium is very crucial for the maintenance of fluid balance in the body. Iron and zinc also varied significantly from 0.34 mg/100 g in *B. aegyptiaca* to 1.46 mg/100 g in *D. mespiliformis*, and from 0.81 mg/100 g in *G. erubescens* to 2.97 mg/100 g in *S. birrea*, respectively. While iron is very vital for the transport of oxygen in the bloodstream and the control of anemia and its attendant effects, zinc is substantially linked with protein synthesis, the catalytic activity of several enzymes, and rapid growth and development during infancy, adolescence, and wound healing [29]. 

### 3.3. Vitamins and Antioxidant Composition 

The vitamin and antioxidant contents of the fruits have been tabulated below (Table 4). The vitamin B3 (niacin) content of the fruits ranged significantly from 146.30 mg/100 g in *G. erubescens* to 932.74 mg/100 g in *S. birrea*. The role of niacin in cellular metabolism and cell longevity cannot be underestimated; thus, niacin plays a critical role in aging [49]. Vitamin C content (mg/100 g) varied significantly from 44.67 mg/100 g in *D. mespiliformis* to 172.49 mg/100 g in *S. birrea*. This vitamin is a crucial antioxidant that enhances non-heme iron transport and absorption, the reduction of folic acid intermediates, and the production of cortisol. Vitamin C is vital in the synthesis of collagen and other connective tissues. Its insufficiency in the body results in the fragility of blood capillaries, gum decay, and scurvy [50,51]. 

Vitamin A is another vital nutrient that is known for its significant role in normal vision, gene expression, growth, and immune functioning [52]. Its content ranged from 0.84 mg/100 g in *B. aegyptiaca* to 2.03 mg/100 g in *S. birrea.* For β-carotene (pro-vitamin A) content, the highest (176.89 mg/100 g) was recorded in *S. birrea* and the lowest (64.84 mg/100 g) was recorded in *B. aegyptiaca*. Beta-carotene enhances the growth of cells and tissues, fortifies the immune system against diseases, and delays aging. Besides that, it helps keep the eye, skin, nails, and hair functioning effectively [53]. The β-carotene values of the fruits serve as a great backup to their vitamin A levels, and therefore could be converted to vitamin A in the body to supplement it.

Polyphenols are known for their free radical scavenging and anticancer properties due to their antioxidant activity [54]. Polyphenol content ranged from 53.39 mg/100 g in *D. mespiliformis* to 111.06 mg/100 g in *S. birrea*, showing the strong potentials of the fruits to arrest free radicals that cause health problems in the body. Flavonoids, another type of antioxidant, possess a strong anti-inflammatory effect, and also protect the body cells against free radical damage, thereby helping control cardiovascular and cancerous problems [55]. Therefore, a range of 94.00 mg/100 g in *D. mespiliformis* to 237.67 mg/100 g in *S. birrea* could be a strong source of fortification against free radical effects.

### 3.4. Antinutrient Composition

The antinutrients (phytate, oxalate, tannin, and saponin) that were reported generally differed significantly (*p <* 0.05) among the fruits (Table 5). The phytate content (mg/g) varied from 0.06 mg/g in *G. erubescens* to 1.82 mg/g in *B. aegyptiaca*. Phytate impedes the absorption of phosphorus, calcium, magnesium, iron, and zinc by forming complexes with them, and reduces amino acid digestibility. This makes these minerals readily unavailable to the body [29]. However, the phytate values obtained in this study were lower than the 10–60 mg/g that has been reported to pose mineral bioavailability problems [56]. The values in this study also averagely fall within the range of 0.37–0.90 mg/g of fruits (guava, mango, orange, and pineapple) that has been recommended elsewhere for diabetics [57]. Despite causing mineral bioavailability problems, phytate is an antioxidant and an anticancer agent.

Oxalate values varied from 0.11 mg/g in *S. birrea* to 0.38 mg/g in *B*. *aegyptiaca*. Oxalate is known to inhibit renal calcium absorption, especially at concentrations of about 45 g/100 g [58]. However, the values obtained in this study are far less than the value that is postulated to be harmful. This suggests that the fruits may not pose any mineral absorption problems if consumed. Tannin content ranged between 0.29 mg/g in *G*. *erubescens* to 0.59 mg/g in *S. birrea*, which is lower than the 1.01 to 7.5 mg/g reported by [31] for similar forest fruits. Tannin has been shown to reduce the palatability of foods with its astringency, inhibit enzyme activities, and reduce protein solubility and digestibility by complexing with them [31]. However, the low tannin content of these fruits may not pose any serious health problems. Saponin content was lowest in *D*. *mespiliformis* (0.22 mg/g), but highest in *S. birrea* (1.05 mg/g). Saponins foam in aqueous solution, and have a characteristic bitter taste that may be imparted to food if it is present in large amounts [59]. Saponins cause the rupturing of red blood cells and trigger nausea and vomiting; nevertheless, they beneficially reduce hypocholesterolemic problems in humans [60]. Research has shown that β-carotene and vitamins A and C are good enhancers of the absorption of minerals such as iron and zinc [61]. Thus, with high contents of β-carotene, and vitamins A and C, antinutrient activity can be inhibited to enhance mineral absorption in the body.

## 4. Conclusions

The study revealed that *Gardenia erubescens, Sclerocarya birrea, Diospyros mespiliformis*, and *Balanites aegyptiaca* are nutritionally sound following their recommendable proximate, mineral, and vitamin/antioxidant values, and contribution to the daily nutrient requirements in humans. The antinutrient contents of the fruits were relatively low, and might not pose any serious health problems, especially with their high concentration of mineral absorption enhancers such as β-carotene and vitamins A and C. Therefore, with the nutritional and antinutritional information provided in this study, it is evident that these forest fruits, which lack public patronage and consumption in the Ghanaian community despite their availability, could be relied on to fight hunger and some nutrient deficiencies. However, due to the seasonality and perishability of the fruits, it would be very expedient to process them into finished and novel food products such as beverages, bakeries, or cookies to ensure extended consumption.

## Figures and Tables

**Table 1:** Names, pictures, and sampling dates of the fruit samples used for the study.

English Name	Scientific Name/Plant	Picture (Fruit)	Sampling Date
Unknown	*Gardenia erubescens*	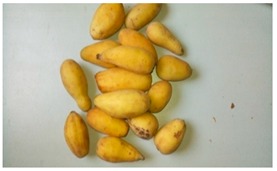	18 March 2018
	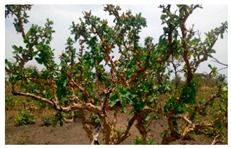		
Marula	*Sclerocarya birrea*	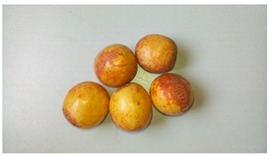	18 March 2018
	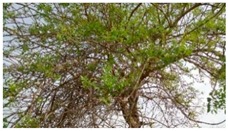		
African ebony	*Diospyros mespiliformis*	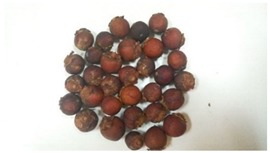	19 March 2018
	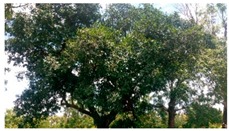		
Desert date	*Balanites aegyptiaca*	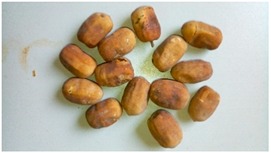	19 March 2018
	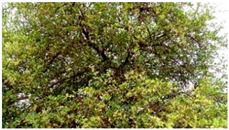		

**Table 2:** Proximate and physicochemical properties of four underutilized forest fruits.

Property	Fruit Samples			
	*G. erubescens*	*S. birrea*	*D. mespiliformis*	*B. aegyptiaca*
Moisture (%) *	70.43 ± 0.24 ^a^	85.84 ± 0.14 ^b^	6.01 ± 0.00 ^#^	18.27 ± 0.10 ^c^
Dry matter (%) *	29.57 ± 0.21 ^a^	14.16 ± 0.38 ^b^	93.99 ± 0.02 ^#^	81.73 ± 0.92 ^c^
Carbohydrate (%)	76.44 ± 0.51 ^a^	61.69 ± 0.69 ^b^	75.23 ± 0.40 ^a^	73.63 ± 0.38 ^c^
Protein (%)	8.83 ± 0.09 ^a^	12.48 ± 0.02 ^b^	10.26 ± 0.01 ^c^	9.19 ± 0.05 ^d^
Ash (%)	3.4 ± 0.20 ^a^	6.37 ± 0.25 ^b^	3.00 ± 0.20 ^a^	4.4 ± 0.20 ^c^
Fat (%)	4.53 ± 0.22 ^a^	9.68 ± 0.17 ^b^	3.51 ± 0.18 ^c^	2.58 ± 0.37 ^d^
Fiber (%)	2.27 ± 0.14 ^a^	4.25 ± 0.17 ^b^	2.00 ± 0.13 ^a^	2.93 ± 0.14 ^c^
Energy (kcal)	381.85 ± 1.61 ^a^	383.81 ± 2.24 ^a^	373.55 ± 1.00 ^b^	354.48 ± 1.93 ^c^
pH	4.64 ± 0.05 ^a^	4.20 ± 0.02 ^b^	5.44 ± 0.04 ^c^	5.35 ± 0.03 ^c^
TSS (°Brix)	1.01 ± 0.43 ^a^	1.21 ± 0.33 ^a^	1.56 ± 0.15 ^a^	1.27 ± 0.61 ^a^
TA (% citric acid)	1.04 ± 0.07 ^a^	1.98 ± 0.31 ^b^	2.04 ± 0.03 ^b^	2.35 ± 0.47 ^b^

Values are mean ± standard deviation. Values with different superscripts in the same row are significantly statistically different. * Determined per 100 g of fresh weight of sample except for *D. mespiliformis*, while the rest were determined per 100 g dry weight of the sample. ^#^ Sample was in the dried state at the time of collection; hence, it was statistically inappropriate to compare its moisture and dry matter with those of the fresh ones. TSS: total soluble solids, TA: titratable acids.

**Table 3:** Mineral composition (mg/100 g dry weight, or dw) of four edible underutilized forest fruits.

Mineral	Fruit Samples			
	*G. erubescens*	*S. birrea*	*D. mespiliformis*	*B. aegyptiaca*
Calcium	6.48 ± 0.04 ^a^	14.95 ± 0.17 ^b^	8.81 ± 0.28 ^c^	10.58 ± 0.11 ^d^
Potassium	133.41 ± 2.37 ^a^	133.02 ± 2.23 ^a^	129.4 ± 1.62 ^a^	131.60 ± 2.69 ^a^
Phosphorus	46.37 ± 0.91 ^a^	103.87 ± 4.32 ^b^	64.78 ± 2.98 ^c^	55.23 ± 0.23 ^d^
Magnesium	168.07 ± 0.44 ^a^	177.69 ± 0.43 ^b^	162.98 ± 0.42 ^c^	166.81 ± 0.51 ^d^
Sodium	2.64 ± 0.04 ^a^	1.75 ± 0.04 ^b^	1.25 ± 0.03 ^c^	1.43 ± 0.05 ^d^
Iron	0.48 ± 0.08 ^a^	0.43 ± 0.02 ^ab^	1.46 ± 0.03 ^c^	0.34 ± 0.01 ^b^
Zinc	0.81 ± 0.02 ^a^	2.97 ± 0.03 ^b^	1.70 ± 0.03 ^c^	2.02 ± 0.03 ^d^

Values are mean ± standard deviation of three replicates. Values in the same row with different superscripts are statistically significantly different.

**Table 4:** Vitamin and antioxidant compositions (mg/100 g) of four underutilized forest fruits.

Nutrient	Fruit Samples			
	*G. erubescens*	*S. birrea*	*D. mespiliformis*	*B. aegyptiaca*
Vitamin B3	146.30 ± 1.48 ^a^	932.74 ± 7.24 ^b^	310.22 ± 8.15 ^c^	227.85 ± 3.80 ^d^
Vitamin C	73.08 ± 2.23 ^a^	172.49 ± 6.01 ^b^	44.67 ± 3.06 ^c^	105.97 ± 2.57 ^d^
Vitamin A	1.17 ± 0.24 ^a^	2.03 ± 0.03 ^b^	1.57 ± 0.32 ^ab^	0.84 ± 0.03 ^ac^
β-carotene	111.01 ± 0.43 ^a^	176.89 ± 9.89 ^b^	89.16 ± 0.77 ^c^	64.84 ± 2.47 ^d^
Polyphenol (GAE)	82.28 ± 8.33 ^a^	111.06 ± 0.10 ^b^	53.39 ± 1.70 ^c^	110.44 ± 1.11 ^b^
Flavonoid (QE)	172.67 ± 8.08 ^a^	237.67 ± 13.50 ^b^	94.00 ± 4.00 ^c^	160.00 ± 6.00 ^a^

Values are mean ± standard deviation of three replicates. Values in the same row with different superscripts are statistically significantly different. GAE—Gallic acid equivalents; QE—Quercetin equivalents.

**Table 5:** Antinutrient content (mg/g) of four underutilized forest fruits.

Fruit	Antinutrient			
	Phytate	Oxalate	Tannin	Saponin
*Gardenia erubescens*	0.06 ± 0.02 ^a^	0.25 ± 0.02 ^a^	0.29 ± 0.09 ^a^	0.72 ± 0.08 ^a^
*Sclerocarya birrea*	0.64 ± 0.04 ^b^	0.11 ± 0.00 ^b^	0.59 ± 0.08 ^b^	1.05 ± 0.06 ^b^
*Diospyros mespiliformis*	0.27 ± 0.02 ^c^	0.14 ± 0.00 ^c^	0.46 ± 0.13 ^ab^	0.22 ± 0.01 ^c^
*Balanites aegyptiaca*	1.82 ± 0.09 ^d^	0.38 ± 0.00 ^d^	0.40 ± 0.03 ^ab^	0.62 ± 0.06 ^a^

Values are mean ± standard deviation of three replicates. Values in the same row with different superscripts are statistically significantly different.
